# Factors Affecting Delayed Recovery from Neutropenia in Patients with Pancreatic Cancer Receiving Gemcitabine plus Nab-Paclitaxel

**DOI:** 10.7150/jca.107359

**Published:** 2025-01-21

**Authors:** Naoko Yoshida, Shungo Imai, Kazuyoshi Kawakami, Takashi Yokokawa, Masashi Nakamura, Takeshi Aoyama, Hisanori Shimizu, Ryoichi Naito, Minori Teramae, Masami Tsuchiya, Hayato Kizaki, Masato Ozaka, Naoki Sasahira, Masakazu Yamaguchi, Satoko Hori

**Affiliations:** 1 Division of Drug Informatics, Keio University Faculty of Pharmacy, Tokyo, Japan.; 2 Department of Pharmacy, Cancer Institute Hospital, Japanese Foundation for Cancer Research, Tokyo, Japan.; 3 Department of Gastroenterology, Cancer Institute Hospital, Japanese Foundation for Cancer Research, Tokyo, Japan.

**Keywords:** gemcitabine, granulocyte colony-stimulating factor, neutropenia, neutrophil, pancreatic neoplasm, risk factor

## Abstract

**Background:** Many studies have identified risk factors for neutropenia associated with various chemotherapies, including gemcitabine plus nab-paclitaxel (GnP). However, few studies have focused on the delayed recovery from neutropenia, which frequently leads to treatment discontinuation. We aimed to examine whether the risk factors for neutropenia affect delayed recovery following development.

**Material and Methods:** Data were collected from patients with pancreatic cancer who received GnP therapy between December 2014 and March 2019 at the Cancer Institute Hospital of the Japanese Foundation for Cancer Research. In the present study, we investigated the following: (1) risk factors for grade 3 or higher neutropenia and (2) factors affecting delayed recovery in patients who developed neutropenia in (1).

**Results:** Of the 638 patients who underwent GnP therapy, 364 developed neutropenia and 29 experienced delayed recovery. Most patients with delayed recovery experienced neutropenia on day 8 of the first course; therefore, we focused on this group. Among the 111 patients who developed neutropenia on day 8 of the first course, 22 experienced delayed recovery after excluding those who received granulocyte-colony stimulating factor. Low baseline neutrophil, platelet, and red blood cell counts were identified as risk factors for neutropenia. Although multivariate analysis could not be conducted due to the limited number of patients, these three factors were also associated with delayed recovery.

**Conclusion:** Low neutrophil, platelet, and red blood cell counts at baseline were identified as risk factors for neutropenia and affecting delayed recovery.

## Introduction

Gemcitabine plus nab-paclitaxel (GnP) is a frontline standard therapy for pancreatic cancer, along with FOLFIRINOX (combination of 5-fluorouracil, leucovorin, irinotecan, and oxaliplatin) 1. A randomized phase II study of modified FOLFIRINOX versus GnP for locally advanced pancreatic cancer in Japan showed that GnP displayed better efficacy in response rate, disease control rate, carbohydrate antigen 19-9 (CA19-9) response, and milder gastrointestinal toxicity. However, myelosuppression and grade 3 or higher neutropenia was predominant in the GnP group (modified FOLFIRINOX vs. GnP: 59.7% vs. 79.4%) 2.

Neutropenia increases susceptibility to several infections, which can sometimes lead to life-threatening conditions, such as febrile neutropenia. In addition, neutropenia frequently leads to treatment discontinuation, and it is difficult to determine when to resume treatment even if the neutrophil count recovers. Therefore, patients may not be able to receive treatment because of neutropenia, despite visiting the hospital, which leads to a burden on patients.

Several previous studies have identified the risk factors for the development of neutropenia associated with various chemotherapies, including GnP therapy 3-10. However, few studies have focused on the recovery of neutrophil counts following its development. Tsushima *et al.*^11^ used data on 27 participants in a phase I study of nab-paclitaxel monotherapy to identify the factors associated with changes in the neutrophil count and also developed a pharmacokinetic and pharmacodynamic model to predict neutropenia.

In several previous studies, white blood cell and platelet counts were identified as risk factors for developing chemotherapy-induced neutropenia, suggesting that they may be good indicators of bone marrow reserve 12-16. Therefore, we hypothesized that patients with these risk factors are likely to experience delayed recovery from neutropenia.

In this study, we aimed to examine whether the risk factors for neutropenia affected delayed recovery following development. Specifically, using electronic medical records, we analyzed the factors affecting delayed recovery in patients who developed neutropenia during GnP therapy.

## Materials and Methods

### Study procedure

To examine whether known risk factors for neutropenia affect delayed recovery following development, we investigated the following: (1) risk factors for grade 3 or higher neutropenia according to the Common Terminology Criteria for Adverse Events (CTCAE) v5.0, in patients with pancreatic cancer who underwent GnP therapy; and (2) factors affecting delayed recovery in patients who developed neutropenia in (1).

### Study design and patients

This study was a retrospective cohort study. Data of 638 patients with pancreatic cancer who underwent at least one cycle of GnP therapy between December 2014 and March 2019 at the Cancer Institute Hospital of the Japanese Foundation for Cancer Research were extracted from electronic medical records.

Patients typically received a 30-min intravenous infusion of nab-paclitaxel (125 mg/m^2^), followed by a 30-min intravenous infusion of gemcitabine (1,000 mg/m^2^) on days 1, 8, and 15, repeated every 4 weeks (Supplementary Figure S1). Laboratory data were obtained before drug administration. In this study, the data were collected up to the sixth cycle.

The inclusion criteria for exploring risk factors for neutropenia were as follows: (1) neutrophil count ≥ 1,000 /μL on day 1 of the first cycle; (2) data on the dose of gemcitabine and nab-paclitaxel administered on day 1 of each cycle were available (dose reductions were allowed from day 1 of the first cycle at the clinician's discretion); (3) laboratory data on day 1 and neutrophil counts on days 8 and 15 of each cycle could be obtained; (4) demographic information, including gender and age could be obtained; (5) no previous GnP therapy; and (6) no granulocyte-colony stimulating factor administration during the observation period.

Additionally, we included patients who met the following criteria for exploring factors affecting delayed recovery from neutropenia: (1) grade 3 or higher neutropenia occurring during the observation period, and (2) laboratory data at the time of neutropenia occurrence and the dose of each anticancer drug on the last administration date before its occurrence.

### Outcome definition

We defined neutropenia as a neutrophil count of < 1,000 /μL during GnP therapy based on grade 3 or higher neutropenia according to CTCAE v5.0. Delayed recovery from neutropenia was defined as a neutrophil count of < 1,000 /μL on the scheduled initial dosing day after development in patients who experienced grade 3 or higher neutropenia during GnP therapy.

### Data collection

We collected the following information: sex, age, body surface area (BSA), performance status, presence of dose reduction, presence of liver metastasis, prior chemotherapy for pancreatic cancer, prior FOLFIRINOX, prior Tegafur Gimeracil Oteracil Potassium, prior Gemcitabine, prior history of diabetes, number of cycles at the development of neutropenia, and laboratory data (red blood cell count [RBC], platelet count [PLT], neutrophil count [Neutr], lymphocyte count [Lymph], serum albumin [ALB], total bilirubin, alkaline phosphatase [ALP], alanine aminotransferase [ALT], urea nitrogen [UN], serum creatinine, and C-reactive protein [CRP]). We also evaluated monocyte count (Mono), although owing to the large number of missing values, we were unable to include it as a variable in the multivariate analysis, but handled it as additional data (i.e., exclusion criterion (3) was not applied). Considering its clinical importance, dose reduction was defined as having occurred when the dose per BSA of either or both drugs was ≤ 80% of the prescribed dose (gemcitabine ≤ 800 mg/m^2^ and nab-paclitaxel ≤ 100 mg/m^2^) in accordance with the guidelines for the appropriate use of medication in Japan 17, 18. If the prescribed dose was over 80%, it was considered as a normal dose. In addition, the details of dose intensity were provided.

Patients receiving GnP therapy underwent blood tests before each dose and the results determined their eligibility for treatment. Laboratory data on day 1 of each cycle were used to explore the risk factors for neutropenia. Additionally, we evaluated laboratory data at the time of neutropenia development to explore the factors affecting delayed recovery. Furthermore, we assessed the range of neutrophil count decline from the last scheduled day of medication administration before development to the date of development.

For laboratory data on days 8 and 15, a one-day deviation from the scheduled date was included in the analysis; however, patients who deviated by more than 2 days were excluded.

### Statistical analysis

Patient characteristics were analyzed using the chi-square test or Fisher's exact test for categorical variables and the Mann-Whitney U test for continuous variables. We compared groups with and without grade 3 or higher neutropenia, as well as groups with and without delayed recovery. Binomial logistic regression analyses were performed using the forced entry method for the variables with p < 0.2 in the univariate analyses (chi-square, Fisher's exact and Mann-Whitney U test). Continuous variables were converted to categorical variables by finding the cutoff value that maximized the Youden Index using receiver operating characteristic (ROC) curve analysis. In the logistic regression analyses, statistical significance was set at p < 0.05, and the odds ratio and 95% confidence interval were calculated for each variable.

IBM SPSS Statistics 29.0 (IBM SPSS Statistics, Chicago, IL, USA) was used for statistical analysis.

### Ethical consideration

This study was approved by the Cancer Institute Hospital of the Japanese Foundation for Cancer Research (No. 2019-1040) and the Ethics Committee of the Keio University Faculty of Pharmacy (No. 200312-9).

## Results

### Patient extraction

Of the 638 patients who underwent GnP therapy, 364 developed neutropenia. Among them, 29 experienced delayed recovery (Supplementary Table S1). Among 29 patients with delayed recovery, 26 (89.7%) developed neutropenia on day 8 of the first cycle.

Therefore, we focused on patients who developed neutropenia on day 8 of the first cycle to explore factors affecting delayed recovery and used the presence of delayed recovery on day 15 of the first cycle as the objective variable. Accordingly, data collected on day 1 of the first cycle were used to explore the risk factors and the objective variable was the development of neutropenia on day 8 of the first cycle.

**Figure 1:**
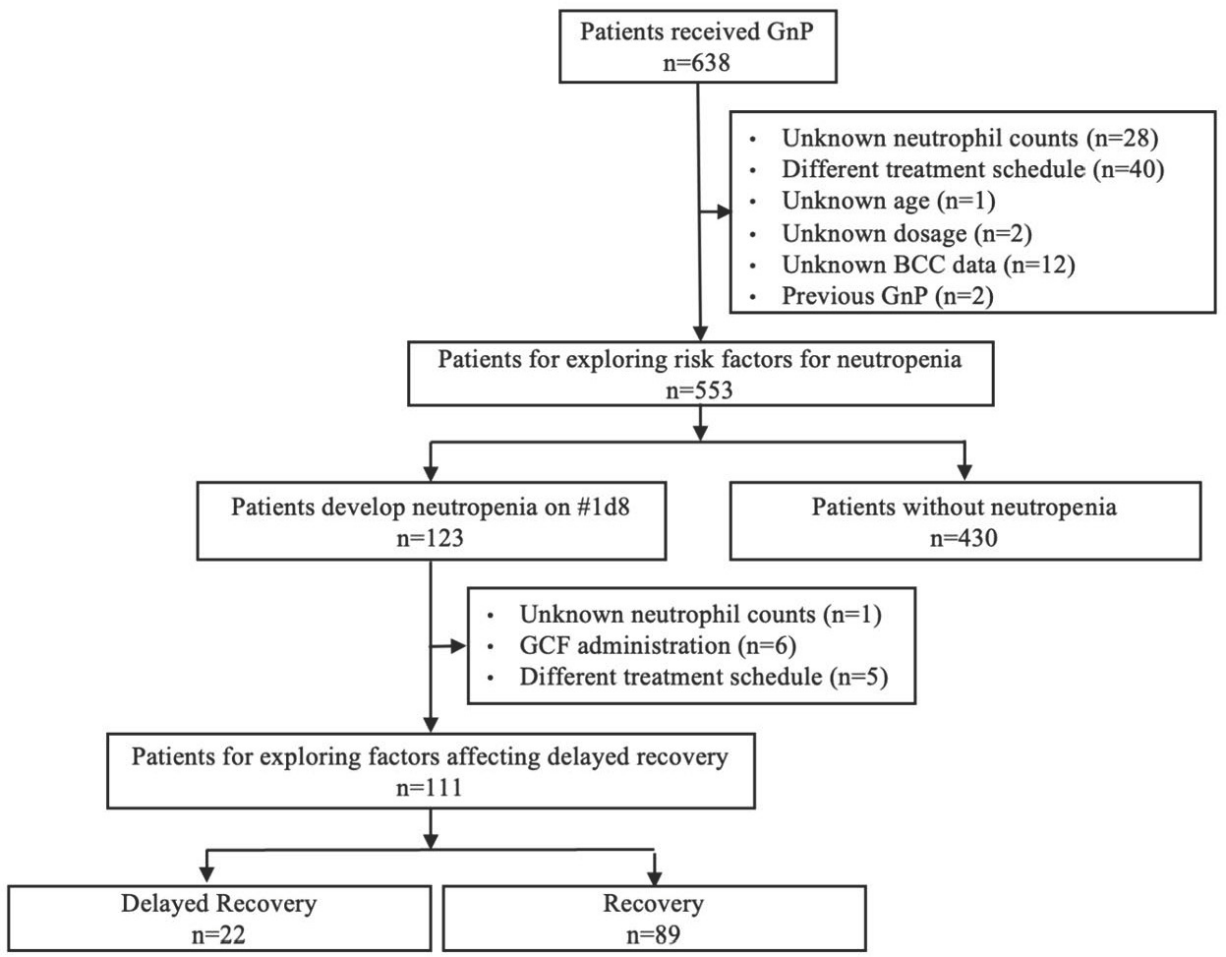
Flow chart for the extraction of patients to be included in the study. GnP, gemcitabine plus nab-paclitaxel; BCC, blood cell count; GCF, granulocyte colony-stimulating factor.

Among 638 patients, 553 were included to explore the risk factors for neutropenia (Figure 1). The incidence of neutropenia on day 8 of the first cycle was 22.2% (123/553). To explore the factors affecting delayed recovery, 111 of the 123 patients were included. The incidence of delayed recovery on day 15 of the first cycle was 19.8% (22/111). Of the 111 patients, 2 patients were administrated gemcitabine and nab-paclitaxel on day 8, of whom one patient experienced delayed recovery.

### Risk factors for neutropenia

Univariate analyses were performed to compare patients who underwent GnP therapy with or without neutropenia on day 8 of the first cycle (Table 1).

The variables with p < 0.2 were sex, age, BSA, dose intensity of gemcitabine, presence of liver metastasis, prior chemotherapy for pancreatic cancer, prior Tegafur Gimeracil Oteracil Potassium, RBC, PLT, Neutr, Lymph, ALP, ALT, UN, and CRP on day 1. In addition, the difference was significant for Mono, although data were missing in 43 patients.

We conducted a multivariate binomial logistic regression analysis with variables that met p < 0.2 criterion in the univariate analysis (Table 2). In addition, we included dose reduction as a variable in order to adjust for the effects of dose intensity. When continuous variables were converted to categorical variables, age < 63, RBC < 3.92 ×10^3^/µL, PLT < 230 ×10^3^/µL, Neutr < 3.60 ×10^3^/µL, and ALP < 322 U/L were identified as significant risk factors of neutropenia (Table 2). When analyzed as continuous variables, a low neutrophil count, low ALP and younger age were identified as significant risk factors (Supplementary Table S2).

### Factors affecting delayed recovery from neutropenia

Univariate analyses were performed to compare the patients with and without delayed recovery among those who developed neutropenia on day 8 of the first cycle (Table 3). Only 22 patients had delayed recovery; therefore, a multivariate analysis could not be conducted 19. Variables with p < 0.05 in the univariate analyses were considered as factors affecting delayed recovery from neutropenia. The variables with p < 0.05 were as follows: low RBC, PLT, Neutr, and high CRP on day 1; low RBC, PLT, and Neutr on day 8; and a small range of neutrophil count declined from days 1 to 8.

### Sensitivity analysis for exploring factors affecting delayed recovery from neutropenia

We conducted chi-square tests using the variables extracted as risk factors for neutropenia (age, RBC, PLT, Neutr, and ALP) and factors affecting delayed recovery (CRP level and range of neutrophil count decline from days 1 to 8). Additionally, Mono was evaluated. To convert these variables into categorical variables, we used the following three cutoff values.

Pattern A: cutoff values set by ROC curve analysis of factors affecting delayed recovery

Pattern B: cutoff values set by ROC curve analysis of risk factors for neutropenia

Pattern C: cutoff values set based on the CTCAE v5.0

In all patterns, Neutr on day 1 was identified as a factor affecting delayed recovery (Supplementary Tables S3-S5). For RBC on day 1, significant differences were observed between patterns A and B. There was no statistically significant difference for PLT on day 1 in pattern B, compared to p < 0.01 in pattern A. Although Mono data were available for only 98 patients, lower Mono was associated with delayed recovery on day 8 of pattern A and day 1 of pattern B. The areas under the ROC curves for Pattern A are shown in Supplementary Figures S2-S11.

## Discussion

### Key finding

This study revealed that the risk factors for neutropenia also affect delayed recovery following development. To our knowledge, this is the first study to explore the factors affecting delayed recovery in patients with pancreatic cancer who develop neutropenia during GnP therapy. The factors commonly identified as risk factors for neutropenia and delayed recovery were low neutrophil, platelet, and red blood cell counts at baseline. In addition, Mono was identified as a potential factor, although the sample size was relatively small.

### Comparison of patients with and without delayed recovery from neutropenia

In the present study, all patients with delayed recovery from neutropenia developed neutropenia on day 8 of each cycle (Supplementary Table S1). Only two patients experienced neutropenia on day 1 of each cycle, and no delayed recovery was observed. For patients who developed neutropenia on day 15, the next scheduled administration date was 14 days later (day 1 of the next cycle) than 7 days later for those who developed neutropenia on days 1 or 8 (Supplementary Figure S1). This may have ensured sufficient time for the neutrophil count to recover to ≥1,000 /μL. Additionally, 89% of the patients experienced delayed recovery during the first course. The dose was reduced in 229 of 503 patients (45.6%) on day 1 of the second cycle, compared with 47 of 553 patients (8.5%) on day 1 of the first cycle. This suggests that clinicians and pharmacists adjusted the dose after the first cycle based on the patient's condition, which helped to prevent neutropenia in subsequent cycles.

### Comparison with risk factors for neutropenia and factors affecting delayed recovery

Low neutrophil and platelet counts have been identified as risk factors for chemotherapy-induced neutropenia in previous studies 15, and similar results were obtained in our study. These variables have been suggested to be good indicators of bone marrow reserve 16. The identification of low neutrophil and platelet counts as factors affecting delayed recovery in our analysis supports the concept that these factors are good indicators of bone marrow reserve. RBC count has not been reported as a risk factor in previous studies. RBCs, platelets and neutrophils are types of blood cells synthesized from common myeloid progenitors. Therefore, the RBC count is thought to be an indicator of hematopoietic capacity of the bone marrow 20. The low baseline blood cell counts observed could have been due to prior chemotherapy for pancreatic cancer. Comparing the area under the ROC curve obtained when the neutrophil, platelet, and RBC counts at baseline were converted to categorical variables, the neutrophil count showed the best discrimination performance (Supplementary Figure S3). This suggests that the neutrophil count is an indicator that reflects bone marrow reserve, which is plausible considering that the half-life of neutrophils ranges from 4 to 18 hours 21-23, and is shorter than that of other blood cells. Mono has been reported as a predictor of chemotherapy-induced neutropenia, and a similar trend was observed in this study as in previous reports 24. Although many patients in this study had missing Mono data, our results suggest that Mono has potential value as a predictor of delayed recovery.

While younger age and low ALP were identified as risk factors in the analysis exploring risk factors for neutropenia, they were not found to be factors affecting delayed recovery.

Many previous studies have identified old age as a risk factor for neutropenia 15. However, this study yielded the opposite results, despite adjusting for dose reduction in the multivariate analysis. This difference may be due to the specific characteristics of the study population, in which GnP therapy was not chosen for older patients who might have a higher risk of neutropenia. Twenty-nine of the 47 patients (61.7%) whose dose was reduced on day 1 of the first cycle were aged 65 years or older. This suggests that clinicians made appropriate treatment decisions in older patients who were at higher risk of neutropenia. Low ALP levels may indicate a decrease in zinc levels 25. Zinc is an important micronutrient in hematopoiesis 26; therefore, patients with decreased zinc levels may be more likely to develop neutropenia. However, Lyman *et al.*^4^ identified high ALP level as a risk factor for neutropenia. Therefore, further studies to clarify the relationship between ALP levels and neutropenia are warranted.

Low CRP was not found to be a risk factor in the analysis exploring risk factors for neutropenia. However, it was identified as a factor affecting delayed recovery. The third quartile was below 0.3 in both groups; therefore, no clinically significant differences were found.

In addition, Tsushima *et al.* 11 reported that albumin is an important predictor of neutropenia in pharmacokinetic and pharmacodynamic models. However, the baseline neutrophil counts of patients in their phase I study were 4,452 and 4,048 for the tri-weekly and weekly regimens, respectively, which differ substantially from the characteristics of our population.

### Limitations

This study had some limitations. First, it was a retrospective study. Data on some important variables including status of operations were not available; therefore, our analyses might not fully reflect the patients' conditions. Second, this study was conducted at a single institution. Therefore, it is necessary to use data from other institutions to examine the generalizability of the results. Third, owing to the small sample size we were unable to conduct a multivariate analysis to explore the factors associated with delayed recovery. These results need to be validated using an independent dataset. However, the factors identified in this study were reproducible to those reported in previous studies regarding neutropenia, suggesting that the findings of this study are valid. Fourth, because the inclusion criterion for this study was a neutrophil count ≥ 1,000 /μL on day 1 of the first cycle, we used the same criteria for recovery. However, the Japanese guidelines for the appropriate use of medication 17 recommend that a neutrophil count ≥ 1,500 /μL be used as a criterion for starting GnP. Therefore, expanding the outcome criteria to several patterns may be useful.

## Conclusion

The risk factors for neutropenia also affected delayed recovery following development. Low neutrophil, platelet, and RBC counts at baseline were identified as risk factors for neutropenia, as well as factors affecting delayed recovery.

## Supplementary Material

Supplementary figures and tables.

## Figures and Tables

**Table 1:** Comparison of patient background factors between neutropenia and non-neutropenia on day 8 of the first cycle

Variable	Neutropenia (n=123)	Non-neutropenia (n=430)	p-value
Female	62 (50.4%)	178 (41.4%)	**0.075**
Age (years)	66.0 (59.0-71.0)	67.0 (61.0-72.0)	**0.151**
BSA	1.56 (1.43-1.67)	1.58 (1.45-1.70)	**0.163**
PS ≥ 1*	15 (12.2%)	72 (16.7%)	0.222
Dose reduction	10 (8.1%)	37 (8.6%)	0.868
Dose intensity of gemcitabine (%)	100 (100-100)	100 (100-100)	0.199
Dose intensity of nab-paclitaxel (%)	100 (100-100)	100 (100-100)	0.203
Liver metastasis	33 (26.8%)	162 (37.7%)	**0.026**
Prior chemotherapy for pancreatic cancer	37 (30.1%)	100 (23.3%)	**0.122**
Prior FOLFIRINOX	7 (5.7%)	36 (8.4%)	0.328
Prior Tegafur Gimeracil Oteracil Potassium	27 (22.0%)	59 (13.7%)	**0.026**
Prior Gemcitabine	3 (2.4%)	15 (3.5%)	0.563
History of diabetes	40 (32.5%)	166 (38.6%)	0.218
Laboratory data on day 1 of the first cycle			
RBC (×10^3^/μL)	3.830 (3.550-4.210)	4.055 (3.735-4.300)	**0.003**
PLT (×10^3^/μL)	193.0 (152.0-233.0)	225.5 (181.0-271.0)	**< 0.001**
Neutr (×10^3^/μL)	2.580 (2.070-3.280)	3.805 (2.985-5.025)	**< 0.001**
Lymph (×10^3^/μL)	1.260 (0.980-1.500)	1.360 (1.050-1.735)	**0.015**
Mono (/μL)	369.8 (298.1-461.8)^ †1^	485.6 (377.6-621.0)^ †2^	**< 0.001**
ALB (g/dL)	3.90 (3.60-4.20)	3.90 (3.60-4.10)	0.653
T-Bil (mg/dL)	0.60 (0.40-0.80)	0.60 (0.40-0.80)	0.665
ALP (U/L)	268.0 (206.0-346.0)	320.5 (232.0-506.0)	**< 0.001**
ALT (U/L)	20.0 (14.0-35.0)	23.0 (15.0-39.0)	**0.127**
UN (U/L)	12.0 (10.0-15.0)	13.0 (10.0-16.0)	**0.106**
Cre (mg/dL)	0.63 (0.54-0.80)	0.65 (0.54-0.78)	0.739
CRP (mg/dL)	0.10 (0.04-0.24)	0.21 (0.07-0.82)	**<0.001**

*Of the 15 patients with PS ≥ 1, three were with PS of 2, one of whom experienced neutropenia. ^†1^ N= 110, ^†2^ N= 400.Values are presented as the median (IQR) or n (%). BSA, body surface area; PS, performance status; FOLFIRINOX, combination of 5-fluorouracil, leucovorin, irinotecan, and oxaliplatin; RBC, red blood cell count; PLT, platelet count; Neutr, neutrophil count; Lymph, lymphocyte count; Mono; monocyte count, ALB, serum albumin; T-Bil, total bilirubin; ALP, alkaline phosphatase; ALT, alanine aminotransferase; UN, urea nitrogen; Cre, serum creatinine; CRP, C-reactive protein

**Table 2:** Multivariate analysis exploring risk factors for neutropenia

Variable	Odds ratio (95% CI)	p-value
Female	1.023 (0.570-1.835)	0.939
Age ≥ 63*	0.418 (0.245-0.712)	**0.001**
BSA ≥ 1.62*	0.861 (0.467-1.585)	0.630
Dose reduction	0.783 (0.337-1.817)	0.569
Liver metastasis	0.776 (0.460-1.308)	0.342
Prior Tegafur Gimeracil Oteracil Potassium	1.656 (0.891-3.079)	0.111
Laboratory data on day 1 of the first cycle		
RBC (×10^3^/μL) < 3.92	2.077 (1.247-3.458)	**0.005**
PLT (×10^3^/μL) < 230*	1.753 (1.055-2.913)	**0.030**
Neutr (×10^3^/μL) < 3.60*	5.083 (2.828-9.135)	**<0.001**
Lymph (×10^3^/μL) < 1.51*	1.675 (0.986-2.846)	0.057
ALP (U/L) ≥ 322*	0.378 (0.216-0.660)	**<0.001**
ALT (U/L) ≥ 17*	0.766 (0.462-1.271)	0.302
UN (U/L) ≥ 15*	0.639 (0.379-1.079)	0.094
CRP (mg/dL) ≥ 0.25*	0.682 (0.392-1.185)	0.174

*These variables were converted to categorical variables by finding the cutoff value that maximized the Youden Index using receiver operating characteristic curve analysis. 95% CI, 95% confidence interval; BSA, body surface area; RBC, red blood cell count; PLT, platelet count; Neutr, neutrophil count; Lymph, lymphocyte count; ALP, alkaline phosphatase; ALT, alanine aminotransferase; UN, urea nitrogen; CRP, C-reactive protein

**Table 3:** Univariate analysis exploring factors affecting delayed recovery from neutropenia

Variables	Delayed recovery (n=22)	Recovery (n=89)	p-value
Female	10 (45.5%)	45 (50.6%)	0.668
Age (years)	68.0 (61.8-74.0)	65.0 (56.5-70.0)	0.136
BSA	1.59 (1.48-1.71)	1.57 (1.43-1.67)	0.375
PS ≥ 1	1 (4.5%)	11 (12.4%)	0.291
Dose reduction	0 (0.0%)	7 (7.9%)	0.174
Liver metastasis	3 (13.6%)	23 (25.8%)	0.226
Prior chemotherapy for pancreatic cancer	7 (31.8%)	25 (28.1%)	0.730
Prior FOLFIRINOX	1 (4.5%)	5 (5.6%)	0.660
Prior Tegafur Gimeracil Oteracil Potassium	6 (27.2%)	18 (20.2%)	0.324
Prior Gemcitabine	0 (0.0%)	2 (2.2%)	0.641
History of diabetes	9 (40.9%)	28 (31.5%)	0.400
Laboratory data on day 1 of the first cycle			
RBC (×10^3^/μL)	3.670 (3.538-3.863)	3.910 (3.570-4.395)	0.006
PLT (×10^3^/μL)	154.0 (140.0-213.0)	209.0 (168.0-246.0)	0.003
Neutr (×10^3^/μL)	1.740 (1.550-2.260)	2.900 (2.305-3.380)	< 0.001
Lymph (×10^3^/μL)	1.320 (0.988-1.410)	1.300 (1.020-1.515)	0.764
Mono (/μL)	312.8 (254.6-384.0)^ †1^	370.0 (302.4-490.0)^ †2^	0.036
ALB (g/dL)	3.95 (3.60-4.13)	4.00 (3.60-4.20)	0.501
T-Bil (mg/dL)	0.65 (0.50-0.80)	0.60 (0.40-0.70)	0.124
ALP (U/L)	253.0 (189.5-312.5)	259.0 (207.5-333.5)	0.513
ALT (U/L)	19.0 (15.0-33.0)	20.0 (14.0-36.0)	0.997
UN (U/L)	13.0 (11.0-15.0)	12.0 (10.0-14.0)	0.347
Cre (mg/dL)	0.66 (0.56-0.78)	0.63 (0.54-0.80)	0.720
CRP (mg/dL)	0.05 (0.02-0.13)	0.10 (0.05-0.24)	0.021
Laboratory data on day 8 of the first cycle			
RBC (×10^3^/μL)	3.505 (3.258-3.768)	3.770 (3.350-4.150)	0.017
PLT (×10^3^/μL)	93.0 (90.8-113.8)	133.0 (108.5-167.0)	< 0.001
Neutr (×10^3^/μL)	0.710 (0.418-0.803)	0.800 (0.620-0.920)	0.008
Lymph (×10^3^/μL)	1.140 (0.780-1.398)	1.100 (0.845-1.295)	0.853
Mono (/μL)	66.3 (43.5-108.0)^ †1^	105.0 (74.8-153.7)^ †2^	0.011
ALB (g/dL)	3.80 (3.48-4.13)	3.90 (3.60-4.10)	0.365
T-Bil (mg/dL)	0.50 (0.40-0.70)	0.50 (0.40-0.60)	0.205
ALP (U/L)	228.5 (159.8-288.8)	253.0 (203.0-330.5)	0.123
ALT (U/L)	19.5 (16.8-27.3)	23.0 (16.0-53.0)	0.273
UN (U/L)	15.0 (12.0-18.3)	13.0 (10.5-16.0)	0.076
Cre (mg/dL)	0.66 (0.55-0.75)	0.62 (0.53-0.77)	0.956
CRP (mg/dL)	0.40 (0.10-0.55)	0.36 (0.15-0.73)	0.450
The range of neutrophil count decline from day 1 to day 8 (×10^3^/μL)	1.235 (0.805-1.665)	2.080 (1.585-2.715)	< 0.001

Values are presented as the median (IQR) or n (%). ^†1^ N= 19, ^†2^ N= 79. BSA, body surface area; PS, performance status; FOLFIRINOX, combination of 5-fluorouracil, leucovorin, irinotecan, and oxaliplatin; RBC, red blood cell count; PLT, platelet count; Neutr, neutrophil count; Lymph, lymphocyte count; Mono; monocyte count, ALB, serum albumin; T-Bil, total bilirubin; ALP, alkaline phosphatase; ALT, alanine aminotransferase; UN, urea nitrogen; Cre, serum creatinine; CRP, C-reactive protein
