# Supplementary material for: Factors Affecting Delayed Recovery from Neutropenia in Patients with Pancreatic Cancer Receiving Gemcitabine plus Nab-Paclitaxel
Source: J Cancer. 2025 Jan 21;16(5):1413–9. doi: 10.7150/jca.107359 (PMC11843245; doi:10.7150/jca.107359)
Supplement: Supplementary file 1 — Supplementary figures and tables. [file jcav16p1413s1.pdf]

Supplementary Material

Supplementary Tables

Supplementary Table S1. Number of patients who developed neutropenia on each administration date and the occurrence of delayed recovery

		Neutropenia Development	Delayed Recovery*
1st cycle	Day 8 (n=611)	131	**26**
	Day 15 (n=484)	136	0
2nd cycle	Day 1 (n=341)	1	0
	Day 8 (n=312)	17	1
	Day 15 (n=293)	21	0
3rd cycle	Day 1 (n=257)	1	0
	Day 8 (n=220)	8	0
	Day 15 (n=223)	12	0
4th cycle	Day 1 (n=211)	–	–
	Day 8 (n=177)	14	0
	Day 15 (n=182)	8	0
5th cycle	Day 1 (n=115)	–	–
	Day 8 (n=177)	5	2
	Day 15 (n=98)	4	0
6th cycle	Day 1 (n=90)	–	–
	Day 8 (n=62)	2	0
	Day 15 (n=82)	4	–
Total		364	29

*Delayed recovery was defined as a neutrophil count of < 1,000 /μL on the scheduled initial dosing day after development in patients who experienced neutropenia.

Supplementary Table S2. Multivariate analysis exploring the risk factors for neutropenia using continuous variables

Variable	Odds ratio (95% CI)	p-value
Female	0.805 (0.428–1.515)	0.502
Age (years)	0.965 (0.939–0.992)	**0.012**
BSA	0.726 (0.114–4.620)	0.734
Dose reduction	1.329 (0.550–3.211)	0.528
Liver metastasis	1.315 (0.779–2.219)	0.305
Prior Tegafur Gimeracil Oteracil Potassium	0.680 (0.367–1.261)	0.221
Laboratory data on day 1 of the first cycle		
RBC ($\times 10^3/\mu L$)	0.974 (0.564–1.680)	0.924
PLT ($\times 10^3/\mu L$)	0.997 (0.993–1.001)	0.206
Neutr ($\times 10^3/\mu L$)	0.397 (0.301–0.524)	**< 0.001**
Lymph ($\times 10^3/\mu L$)	0.804 (0.474–1.365)	0.419
ALP (U/L)	0.998 (0.997–1.000)	**0.040**
ALT (U/L)	0.998 (0.984–1.012)	0.753
UN (U/L)	0.987 (0.931–1.047)	0.671
CRP (mg/dL)	0.765 (0.503–1.164)	0.211

95% CI, 95% confidence interval; BSA, body surface area; RBC, red blood cell count; PLT, platelet count; Neutr, neutrophil count; Lymph, lymphocyte count; ALP, alkaline phosphatase; ALT, alanine aminotransferase; UN, urea nitrogen; CRP, C-reactive protein

Supplementary Table S3. Chi-square test for exploring factors affecting delayed recovery: Pattern A

Variables	Delayed recovery (n=22)	Recovery (n=89)	p-value
Age ≥ 67	14 (63.6%)	39(43.8%)	0.096
Laboratory data on day 1 of the first cycle			
RBC (×10^3/μL) < 3.98	20 (90.9%)	45 (50.6%)	**< 0.001**
PLT (×10^3/μL) < 164	15 (68.2%)	17 (19.1%)	**< 0.001**
Neutr (×10^3/μL) < 2.06	16 (72.7%)	11 (12.4%)	**< 0.001**
Mono (/μL) < 359.3	11 (57.9%)[†1]	38 (48.1%)[†2]	0.443
ALP (U/L) ≥ 196	4 (18.2%)	23 (25.8%)	0.453
CRP (mg/dL) ≥ 0.08	7 (31.8%)	56 (62.9%)	**0.008**
Laboratory data on day 8 of the first cycle			
RBC (×10^3/μL) < 3.87	21 (95.5%)	55 (61.8%)	**0.002**
PLT (×10^3/μL) < 98	14 (63.6%)	13 (14.6%)	**< 0.001**
Neutr (×10^3/μL) < 0.88	21 (95.5%)	58 (65.2%)	**0.005**
Mono (/μL) < 104.1	14 (73.7%)[†1]	38 (48.1%)[†2]	**0.045**
ALP (U/L) ≥ 295	4 (18.2%)	33 (37.1%)	0.092
CRP (mg/dL) ≥ 0.96	0 (0.0%)	17 (19.1%)	**0.026**
The range of decrease in number of neutrophils (×10^3/μL) ≥ 1.38	7 (31.8%)	76 (85.4%)	**< 0.001**

Pattern A: The variables were converted to categorical variables using cut-off values by ROC curve analysis in the analyses of factors affecting delayed recovery. [†1] N= 19, [†2] N= 79.
RBC, red blood cell; PLT, platelet count; Neutr, neutrophil count; Mono, monocyte count; ALP, alkaline phosphatase; CRP, C-reactive protein

Supplementary Table S4. Chi-square test for exploring factors affecting delayed recovery; Pattern B

Variable	Delayed recovery (n=22)	Recovery (n=89)	p-value
Age ≥ 63	16 (72.7%)	53 (59.6%)	0.254
Laboratory data on day 1 of the first cycle			
RBC (×10^3/μL) < 3.92	17 (77.3%)	45 (50.6%)	**0.024**
PLT (×10^3/μL) < 230	19 (86.4%)	59 (66.3%)	0.065
Neutr (×10^3/μL) < 3.60	22 (100%)	71 (79.8%)	**0.021**
Mono (/μL) < 418.5	18 (94.7%)[†1]	50 (63.3%)[†2]	**0.076**
ALP (U/L) ≥ 196	15 (68.2%)	73 (82.0%)	0.152
CRP (mg/dL) ≥ 0.25	2 (9.1%)	21 (23.6%)	0.133

Pattern B: Variables were converted to categorical variables using cutoff values by ROC curve analysis in the analyses of risk factors for neutropenia. [†1] N= 19, [†2] N= 79.

RBC, red blood cell count; PLT, platelet count; Neutr, neutrophil count; Mono, monocyte count; ALP, alkaline phosphatase; CRP, C-reactive protein

Supplementary Table S5. Chi-square test for exploring factors affecting delayed recovery; Pattern C

Variable	Delayed recovery (n=22)	Recovery (n=89)	p-value
Laboratory data on day 1 of the first cycle			
Neutr ($\times 10^3/\mu L$) < 1.50	3 (13.6%)	2 (2.2%)	**0.021**
Laboratory data on day 8 of the first cycle			
Neutr ($\times 10^3/\mu L$) < 0.50	8 (36.4%)	9 (10.1%)	**0.002**

Pattern C: Neutrophil count less than 1,500/μL corresponds to grade 1 neutropenia in CTCAE v.5, and less than 500/μL corresponds to grade 4.
Neutr, neutrophil count

Supplementary Figures

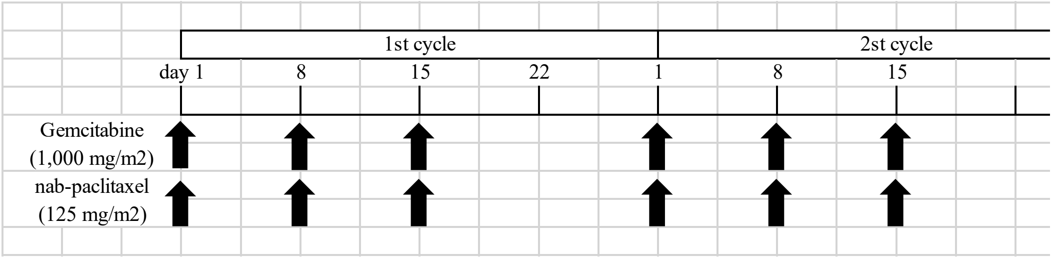

Supplementary Figure S1. Standard treatment schedule for gemcitabine plus nab-paclitaxel Laboratory data were obtained before drug administration

Supplementary Figures S2-11: Receiver operating characteristic curve for the analyses of factors affecting delayed recovery from neutropenia (Pattern A)

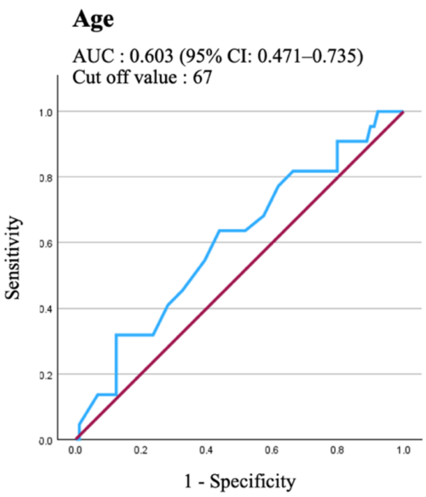

Supplementary Figure S2: A receiver operating characteristic curve of age
AUC, area under the curve; CI, confidence interval; N= 111.

Day 1 Blood cell count data

Neutr
AUC : 0.859 (95% CI: 0.787–0.932) Cut off value : 3.98
PLT
AUC : 0.705 (95% CI: 0.579–0.830) Cut off value : 164
RBC
AUC : 0.690 (95% CI: 0.588–0.792) Cut off value : 2.06

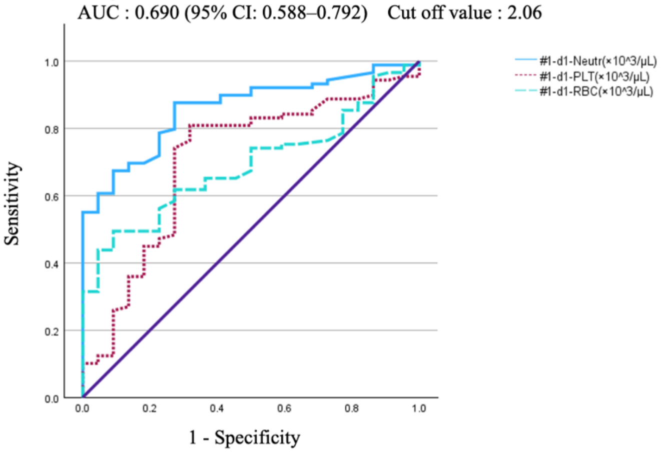

Supplementary Figure S3: A receiver operating characteristic curve of blood cell count on day 1 of the first cycle
RBC, red blood cell count; PLT, platelet count; Neutr, neutrophil count; AUC, area under the curve; CI, confidence interval; N= 111.

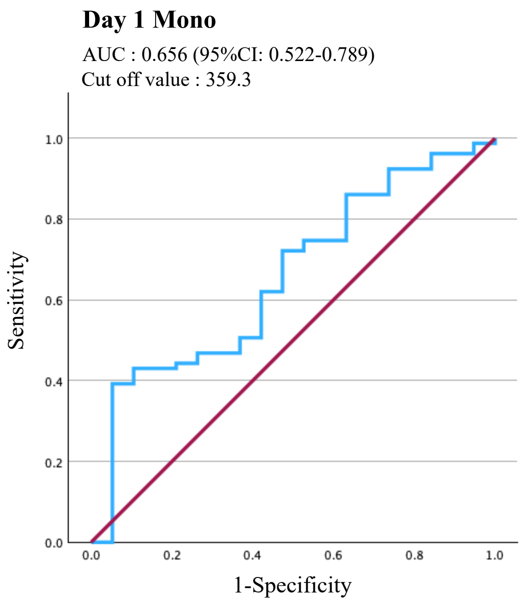

Supplementary Figure S4: A receiver operating characteristic curve of monocyte count on day 1 of the first cycle

Mono, monocyte count; AUC, area under the curve; CI, confidence interval; N= 98.

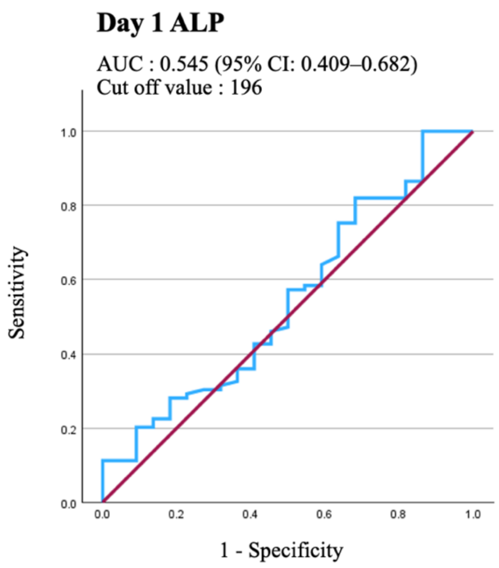

Supplementary Figure S5: A receiver operating characteristic curve of alkaline phosphatase on day 1 of the first cycle
ALP, alkaline phosphatase; AUC, area under the curve; CI, confidence interval; N= 111.

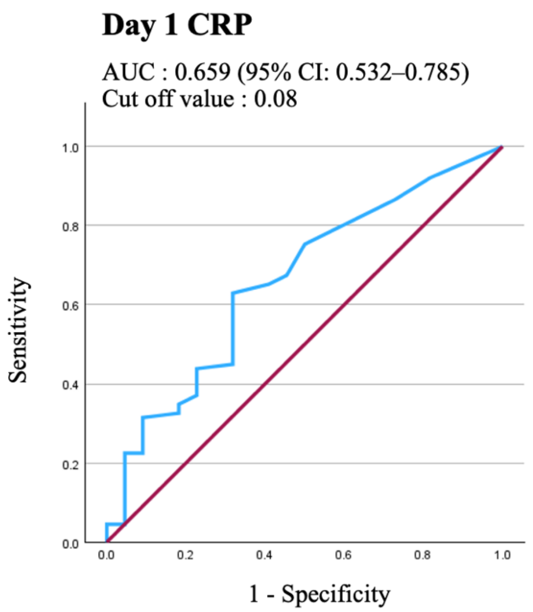

Supplementary Figure S6: A receiver operating characteristic curve of C-reactive protein on day 1 of the first cycle

CRP, C-reactive protein; AUC, area under the curve; CI, confidence interval; N= 111.

Day 8 Blood cell count data

Neutr
AUC : 0.684 (95% CI: 0.567–0.800) Cut off value : 0.88
PLT
AUC : 0.770 (95% CI: 0.663–0.877) Cut off value : 98
RBC
AUC : 0.665 (95% CI: 0.557–0.772) Cut off value : 3.87

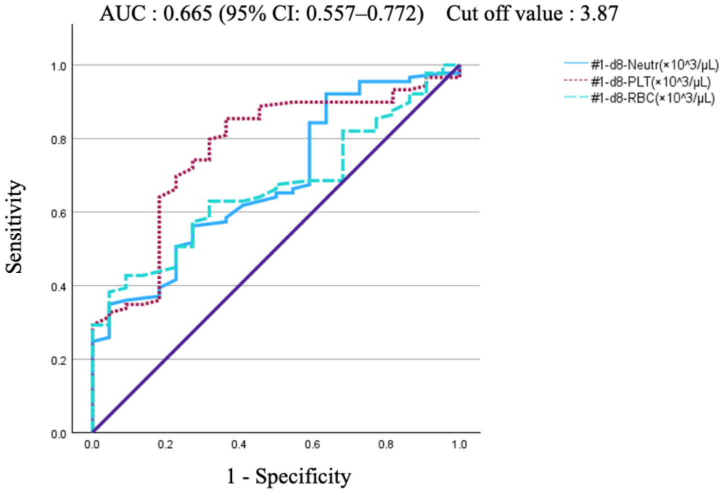

Supplementary Figure S7: A receiver operating characteristic curve of blood cell count on day 8 of the first course

RBC, red blood cell count; PLT, platelet count; Neutr, neutrophil count; AUC, area under the curve; CI, confidence interval; N= 111.

Day 8 Mono

AUC : 0.689 (95%CI: 0.547-0.831)

Cut off value : 104.1

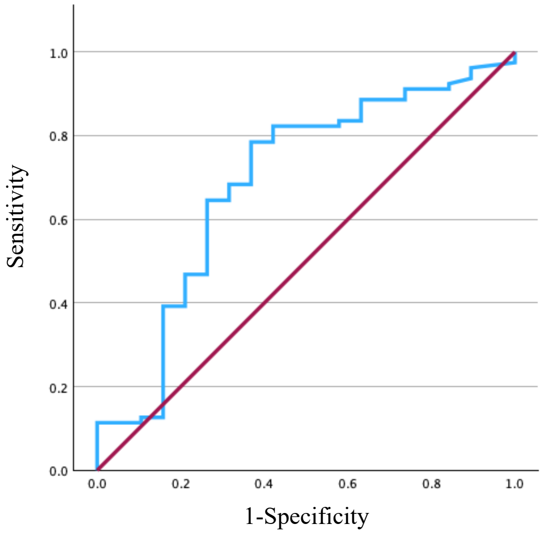

Supplementary Figure S8: A receiver operating characteristic curve of monocyte count on day 8 of the first cycle

Mono, monocyte count; AUC, area under the curve; CI, confidence interval; N= 98.

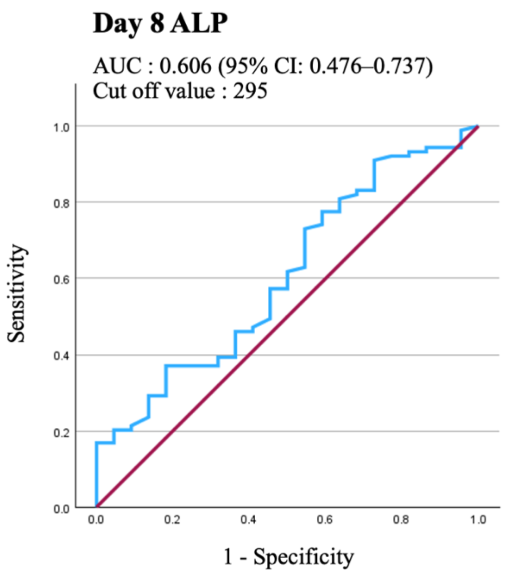

Supplementary Figure S9: A receiver operating characteristic curve of alkaline phosphatase on day 8 of the first cycle

ALP, alkaline phosphatase; AUC, area under the curve; CI, confidence interval; N= 111.

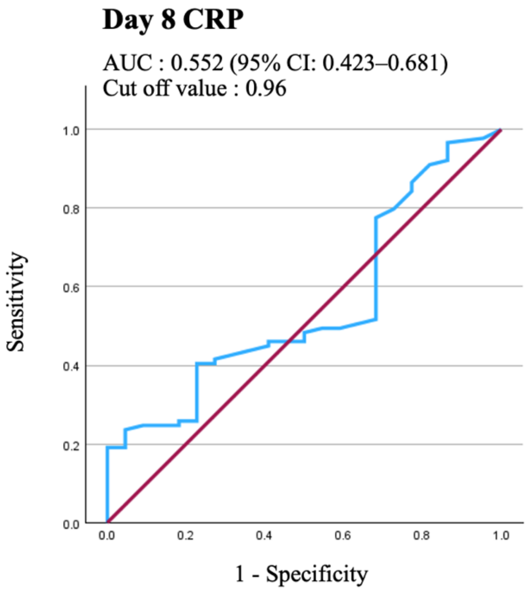

Supplementary Figure S10: A receiver operating characteristic curve of C-reactive protein on day 8 of the first cycle

CRP, C-reactive protein; AUC, area under the curve; CI, confidence interval; N= 111.

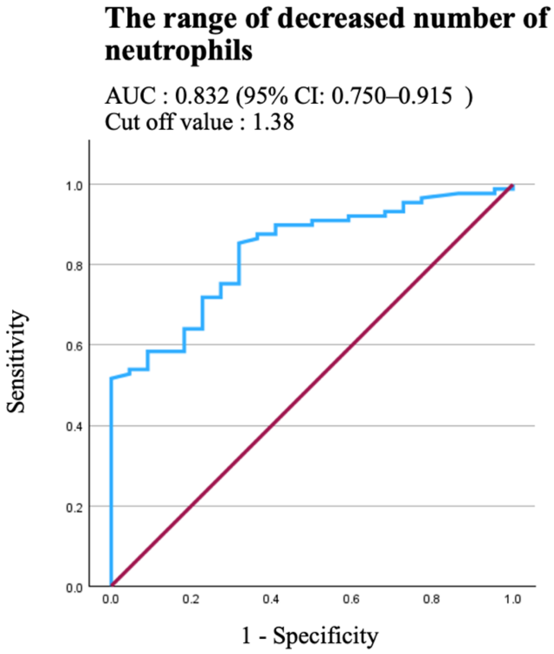

Supplementary Figure S11: A receiver operating characteristic curve of the range of decreased numbers of neutrophils
AUC, area under the curve; CI, confidence interval; N= 111.
